# Multidimensional Oncological Frailty Scale (MOFS): A New Quick-To-Use Tool for Detecting Frailty and Stratifying Risk in Older Patients with Cancer—Development and Validation Pilot Study

**DOI:** 10.3390/cancers15051553

**Published:** 2023-03-01

**Authors:** Riccardo Franchi, Chukwuma Okoye, Rachele Antognoli, Igino Maria Pompilii, Irene Taverni, Tommaso Landi, Matteo Ghilli, Manuela Roncella, Valeria Calsolaro, Fabio Monzani

**Affiliations:** 1 Geriatrics Unit, Department of Clinical & Experimental Medicine, University Hospital of Pisa, Via Savi 10, 56126 Pisa, Italy; 2 Aging Research Center, Department of Neurobiology, Karolinska Institutet, 17165 Stockholm, Sweden; 3 Breast Cancer Centre, University Hospital of Pisa, Via Savi 10, 56126 Pisa, Italy

**Keywords:** frailty detection, geriatric oncology, risk stratification

## Abstract

**Simple Summary:**

In the present pilot study, we developed and validated a new accurate, quick-to-use screening tool for stratifying frailty and the risk of mortality in oncological geriatric patients. Frailty detection with comprehensive geriatric assessment (CGA) is of pivotal importance in older cancer patients to avoid over- or under-treatment with chemotherapy and to detect those at increased risk for poor outcomes. Several tools have been developed to capture the complexity of frailty, but only a few were explicitly conceived for older patients with cancer. In this study, we consecutively enrolled older patients with breast cancer during the preoperative evaluation as the development cohort. We evaluated seventy patients with different types of cancer admitted to our OncoGeriatric Clinic for the validation cohort. We then evaluated the relationship between MPI and CGA items, and we finally realized a screening tool based on the combination of the significant variables, now called the Multidimensional Oncological Frailty Scale (MOFS).

**Abstract:**

Background: Frailty detection with comprehensive geriatric assessment (CGA) is of pivotal importance in older patients with cancer to avoid over- or under-treatment and to detect those at increased risk for poor outcomes. Several tools have been developed to capture the complexity of frailty, but only a few were explicitly conceived for older adults with cancer. The study aimed at developing and validating a multidimensional, easy-to-use diagnostic tool for early-risk stratification in patients with cancer, called the Multidimensional Oncological Frailty Scale (MOFS). Methods: In this single-center prospective study, we consecutively enrolled 163 older women (age ≥ 75 years) with breast cancer, screened with a G8 score ≤ 14 during the outpatient preoperative evaluation at our breast centre, as the development cohort. Seventy patients with different types of cancer admitted to our OncoGeriatric Clinic served as the validation cohort. Using stepwise linear regression analysis, we evaluated the relationship between Multidimensional Prognostic Index (MPI) and CGA items, and, finally, realized a screening tool based on the combination of the significant variables. Results: The mean age of the study population was 80.4 ± 5.8 years, while the mean age of the validation cohort was 78.6 ± 6.6 years [42 women (60%)]. A composite model of the Clinical Frailty Scale, G8, and hand grip strength test showed a strong correlation with MPI (R= −0.712, *p* < 0.001). The MOFS accuracy in the prediction of mortality was optimal in both the development and the validation cohorts (AUC 0.82 and 0.87; *p* < 0.001 and 0.003, respectively). Conclusion: MOFS represents a new, accurate, quick-to-use frailty screening tool for stratifying the risk of mortality in geriatric cancer patients.

## 1. Introduction

A significant proportion of cancer diagnoses occur in older adults and approximately 80% of cancer deaths each year account for this population [1]. By 2035, it is estimated that 60% of all new cancer diagnoses will be attributable to geriatric patients [2].

In the elderly, cancer can significantly impact the course of pre-existing comorbidities due to the onset of physiological, psychological, and functional changes [3]. This burden of vulnerability predisposes geriatric patients with cancer to an increased risk of poor outcomes and adverse events [4]. Considering that not only the neoplastic disease but also its pharmacological and surgical therapy can represent significant stress triggers that impact the patient’s physiological reserve, the precise and punctual definition of risk exposure is essential for establishing individualized treatment pathways and adequate follow-up algorithms [5].

Nonetheless, the preliminary assessment of treatment’s associated risks is difficult and challenging due to the significant, albeit asymptomatic, differences in physical reserve [6] and the lack of a standardized protocol regarding the prognostic evaluation of older patients with cancer.

Frailty assessment is a proven excellent evaluation tool of physiological reserves [7] since more than half of elderly cancer patients are affected by a condition of frailty or prefrailty [8], with a consequently higher risk of developing adverse outcomes [9,10,11].

Moreover, frailty evaluation was progressively recognized as a good predictor of postoperative complications, chemotherapy intolerance, disease progression, and death in cancer patients [12]; its evaluation is, therefore, recommended to assess eligibility for cancer treatment [13].

Several tools have been developed to capture the complexity of frailty. Still, only a few were explicitly conceived for older adults with cancer, such as the G8 questionnaire as a screening tool [14] and Onco-MPI [15] for extensive assessment. Comprehensive Geriatric Assessment (CGA) with Multidimensional Prognostic Index (MPI) is a multidimensional geriatric model focused on the various patient domains. It represents a benchmark for determining global vulnerability in older adults by assessing their functional, psychosocial, cognitive, and nutritional status in order to develop a coordinated and integrated treatment plan and subsequently adequate long-term follow-up [16,17]. Nonetheless, MPI includes functional and cognitive tests that are recognized to be time-consuming and require the presence of a skilled geriatrician to be correctly performed [15].

In this regard, the International Society of Geriatric Oncology (SIOG) recommends a two-stage approach starting with a preliminary screening useful for identifying individuals at greater risk of frailty, who could, therefore, derive a significant benefit from performing a complete CGA [18].

Among various screening tools tested, the Geriatric 8 questionnaire (G8) has been validated in the literature as the most reliable screening tool [14,19,20] to identify patients who may benefit from an extensive evaluation [21]. However, despite its established screening value, evidence concerning its predictive role for overall survival and complication rate is still debated [22,23].

This study aimed at developing and validating a multidimensional, easy-to-use diagnostic tool for early-risk stratification in older patients with cancer, called Multidimensional Oncological Frailty Scale (MOFS).

## 2. Materials and Methods

In this single-center prospective study, two cohorts of very old patients were included. In the first cohort (development cohort), we consecutively enrolled 163 older female patients (age ≥ 75 years) with breast cancer, screened for CGA evaluation by the G8 questionnaire [14] during the outpatient preoperative evaluation at our breast center from June 2020 to June 2021 for developing MOFS. The inclusion criteria were as follows: (1) age ≥ 75 years; (2) impaired G8 score (≤14); (3) eligibility for surgical treatment with or without neoadjuvant or adjuvant chemo/radiotherapy; (4) ability to provide informed consent; (5) ability to perform a hand grip test.

The exclusion criteria were as follows: (1) terminally ill patients with Clinical Frailty Scale (CFS) score equal to 9 suggestive of a life expectancy fewer than 6 months, supported by the treating oncologist with prescription of palliative therapy only; (2) end-stage liver disease (namely, patients with abnormal liver function and the coexistence of one or more criteria of end-stage liver disease (ESLD), such as ascites, variceal bleeding, hepatic encephalopathy, or renal impairment) and end-stage kidney disease (patients with estimated glomerular filtration rate (EGFR) values less than 15 mL/min or need for hemodialysis; (3) inability to provide informed consent; (4) inability to perform a hand grip test.

The G8 questionnaire is a screening tool commonly used in oncology practice consisting of eight items (indication of age and seven selected from MNA) for identifying geriatric oncological patients who could benefit from CGA. The G8 score ranges from 0 (heavily impaired) to 17 (not at all impaired); a score ≤14 is defined as impaired.

In the second cohort (validation cohort), we evaluated 70 patients admitted to our OncoGeriatric Clinic with different types of cancer at first diagnosis eligible for medical/surgical treatment to perform CGA and to validate the efficacy and accuracy of MOFS as a frailty screening tool and potential risk prognostic scale. Subsequently, telephone follow-up was performed at 12 months, recording the mortality rate.

All the patients underwent anamnestic evaluation, physical examination, and a comprehensive geriatric assessment (CGA) [24], including the Multidimensional Prognostic Index (MPI) [25] and the CFS [26], a judgment-based frailty tool that evaluates specific domains, including comorbidity, physical function, and cognition to generate a frailty score ranging from 1 (very fit) to 9 (terminally ill).

MPI is a direct product of the CGA, which uses a mathematical algorithm including information about 8 domains: level of autonomy in terms of independence in the performance of basic (ADL) [27] and instrumental (IADL) [28] activities of daily living, cognitive status evaluation using the Short Portable Mental Status Questionnaire (SPMSQ) [29], nutritional status assessment through the Mini Nutritional Assessment-Short Form (MNA-SF) [30], mobility impairment and consequent risk of developing pressure sores using the Exton-Smith Scale (ESS) [31], multimorbidity evaluated by the Cumulative Illness Rating Scale (CIRS) [32], polypharmacy taken, and cohabitation status including household composition, home services, and institutionalization.

Each domain scored from 0 to 1 point (0, no problems; 0.5, minor problems; and 1, major problems). The sum of the calculated scores from each domain was divided by the eight domains to obtain a final MPI score from 0 to 1. MPI identifies three grades of risk: low-risk MPI 1 (0.0–0.33); moderate-risk MPI 2 (0.34–0.66) and severe-risk MPI 3 (0.67–1.0).

Furthermore, the risk of malnutrition was assessed by calculating Body Mass Index (BMI). Functional capabilities and physical performance were evaluated through SPPB and HGS.

The SPPB [33] measures physical function using 3 components: usual gait speed over 4 m, time to complete 5 chair rises, and standing balance with progressively narrow base of support. Each component is scored on a 0–4 scale and summed for an overall range of 0–12. The hand grip strength (HGS) test was performed using a hand dynamometer with the dominant hand. HGS is a simple measure of strength and may be utilized as a marker of muscle function and mobility. Participants were seated with shoulder adducted, elbow flexed to 90 degrees, and forearm and wrist neutral. The highest score of three consecutive measurements was recorded. The cut-off points of <20 kg in women and <30 kg in men have been identified to detect patients at risk for sarcopenia.

The study complied with the Declaration of Helsinki and was approved by the local Ethics Committee (Mam_Onco_Ger—6 May 2020). Each patient provided written informed consent to participate in this study.

### Statistical Analysis

Statistical analysis was performed with IBM SPSS Statistic (IBM SPSS Statistic version 27.0 lnk IBM Corporation and its licensor 1989–2020) and RStudio (RStudio Team: Integrated Development for R. RStudio, PBC, Boston, MA, USA). Continuous variables were presented as mean and standard deviation (SD), ordinal variables as median and interquartile range (IQR), and categorical variables as percentage (%). The Mann–Whitney U test and chi-square test were used for multiple comparisons. Using a backward stepwise linear regression analysis, we evaluated the relationship between MPI and CGA items. The probability for removal of variables in the model was set at *p* = 0.10 or higher. Finally, we realized a screening tool based on a combination model of the significant variables using the standardized coefficient of correlation. As sensitivity analysis, bootstrapping method with 1000 samples was applied. As secondary analysis, the Pearson correlation was performed to verify the relationship between MPI and our frailty screening tool called MOFS. A multivariate logistic regression was finally performed to evaluate the association between MOFS and 1-year mortality in both cohorts. The covariates included were age in the development cohort, and age and sex in the validation cohort. Estimated odds ratios (ORs) with 95% confidence intervals (CIs) and area under the curve (AUC) were obtained. The DeLong test [34] was assessed to compare MOFS and G8 AUCs. In order to estimate the threshold value most associated with mortality, a ROC curve was calculated in both cohorts between MOFS score compared to 1-year mortality.

## 3. Results

Clinical characteristics of the development and the validation cohorts are summarized in Table 1 and Table 2, respectively. The mean age of the development cohort was 80.4 ± 5.8 years while the mean age of the validation cohort was 78.6 ± 6.6 years (42 women (60%)).

The cancer types of the validation cohort were as follows: 17 colorectal (24.2%), 12 ovarian (17.1%), 11 lung (15.7%), 10 breast (14.2%), 5 gastric (7.1%), 4 pancreatic (5.7%), 3 hematological (4.3%), 3 genitourinary (4.3%), 2 esophageal (2.9%), 1 sarcoma (1.5%), 1 melanoma (1.5%) and 1 GIST (1.5%) (Table 3).

**Figure 1:**
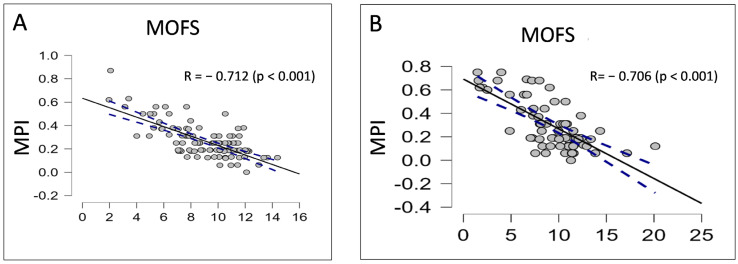
Correlation plot showing relationship between MOFS and MPI in development (**A**) and validation cohort (**B**).

Through multivariate stepwise regression analysis, after extensive adjustment, a composite model of the Clinical Frailty Scale, G8 questionnaire, and hand grip strength test showed a strong correlation with MPI (Pearson’s R 0.742, *p* = 0.003). MOFS was then derived by using each item’s inverse β standardized coefficient (CFS X—0.3; G8 X 0.3; hand grip X 0.3) to obtain a score with a positive value (Figure 1). As compared with G8, MOFS showed a higher correlation with frailty (MOFS Pearson’s R: −0.712; G8 Pearson’s R: −0.426), yielding a high diagnostic performance in detecting frailty, stronger than that obtained by G8 alone (AUC 0.94 vs. 0.85, *p* < 0.001 by the DeLong Test).

**Figure 2:**
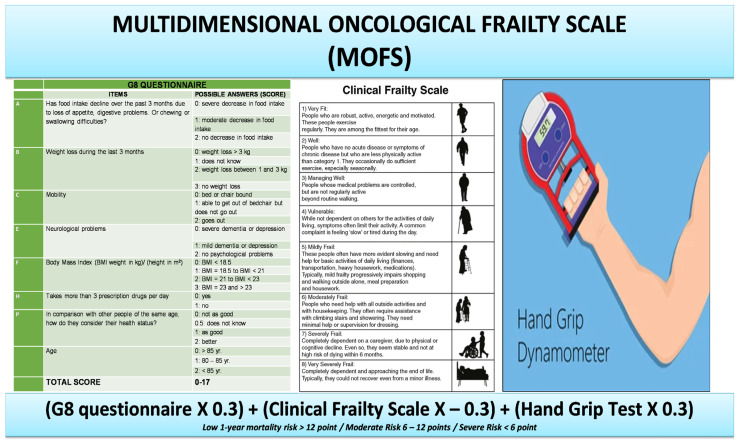
Multidimensional Oncological Frailty Scale (MOFS).

The preliminary analysis of our pilot study shows that MOFS, similarly to MPI, identifies three grades of risk: low-risk (MOFS score >12); moderate risk (between 6 and 12) and severe risk (<6) (Figure 2).

The mean time to perform the CGA and the MPI was 40 ± 1.2 min in the development cohort and 42 ± 1.4 min in the validation cohort. The mean time to perform the G8 questionnaire was 9 ± 1.1 min in the development cohort and 6 ± 1.3 min in the validation cohort, whereas the mean time to perform MOFS in the validation cohort was 8.6 ± 1.5 min. The 12-month mortality rate was 12% (21 patients) in the development cohort, and 21% (15 patients) in the validation cohort. Deceased patients in the development cohort showed a higher degree of functional impairment on most evaluation scales as compared to those alive at follow-up; in detail, they had a higher degree of frailty [median CFS 4.5 (IQR 2.5) vs. 3 (IQR 2), respectively; *p* = 0.006], a more impaired G8 score [median G8 9 (IQR 1.625) vs. 13 (IQR 3), respectively; *p* < 0.001], a lower nutritional status [median MNA-SF 11 (IQR 3.5) vs. 12 (IQR 3), respectively, *p* = 0.004]; a lower physical performance [median SPPB 3 (IQR 6) vs. 8 (IQR 7), respectively, *p* = 0.006], a lower hand strength [mean HGS 14.06 (SD 4.82) vs. 18.15 (SD 0.15), respectively, *p* = 0.02] and a higher MPI value [mean MPI 0.41 (SD 0.20) vs. 0.26 (SD 0.14), respectively, *p* = 0.003] compared to their counterparts. In contrast, no relevant differences were found among chronological age, disability degree (ADL and IADL), cognitive impairment, mood disorder, comorbidity burden, number of drugs, and body weight.

**Figure 3:**
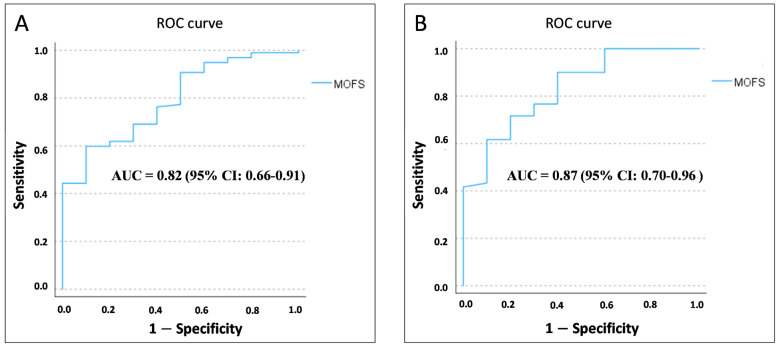
ROC curve of MOFS compared to 1-year mortality in development (**A**) and validation cohort (**B**).

Deceased patients in the validation cohort were more frequently females than males (70% vs. 30 %, *p* = 0.03) and showed a higher impairment at most evaluation scales as compared to those still alive at follow-up; in detail, they had a higher degree of disability both at basic [median BADL 5 (IQR 4.75) vs. 6 (IQR 1), respectively; *p* = 0.003] and instrumental activities of daily living [median IADL 0 (IQR 7.25) vs. 8 (IQR 2), respectively; *p* < 0.001]; a higher degree of frailty [median CFS 6.5 (IQR 1.750) vs. 3 (IQR 2), respectively; *p* < 0.001]; an impaired G8 score [median G8: 7.25 (IQR = 3.750) vs. 12.75 (IQR 4.625), respectively; *p* < 0.001], a lower nutritional status [median MNA-SF 4 (IQR 2.5) vs. 11 (IQR 3.5), respectively; *p* < 0.001], a more impaired physical performance [median SPPB 2 (IQR 5) vs. 7 (IQR 6), respectively; *p* = 0.005], a lower muscle strength [mean HGS 14.7 (SD 6.69) vs. 20.24 (SD 6.86), respectively; *p* = 0.02], a greater burden of comorbidities [median CIRS 3.5 (IQR 1.75) vs. 2 (IQR 2), respectively; *p* = 0.02], a higher degree of cognitive impairment [median SPSMQ 5 (IQR 5) vs. 1 (IQR 1), respectively; *p* < 0.001], and a higher MPI value [mean MPI 0.57 (SD 0.18) vs. 0.25 (SD 0.17), respectively; *p* < 0.001] compared to counterparts. At the same time, no significant differences were found regarding chronological age and the number of drugs. The diagnostic accuracy of MOFS in the prediction of mortality was optimal in both the development and the validation cohort, as shown in Figure 3 (AUC 0.82 and 0.87, respectively; *p* < 0.001 and 0.003), with a cut-off value for high mortality risk lower than 6 (Sensitivity 86%, Specificity 71%).

## 4. Discussion

In our cohort of very old patients, the new prognostic screening tool, now called MOFS, emerged as an accurate, quick-to-use, and easy-to-perform instrument useful for detecting and stratifying frailty with good potential in predicting mortality risk in oncological geriatric patients. Conceiving MOFS, we chose to select the development cohort in a peculiar and homogeneous oncological setting composed of patients of the same sex affected by the same type of cancer, which has a significant prevalence in both the general and geriatric populations, characterized by a good short–medium-term prognosis and a low-impact surgery, in order to investigate more precisely the frailty degree and the silent differences in physical reserve present in the older adults. Conversely, to validate MOFS, we chose to select the validation cohort in a heterogeneous setting characterized by patients of both sexes with different types of cancer and, consequently, a different prognosis and surgical burden. In both cohorts, we observed the same diagnostic accuracy of MOFS in detecting and stratifying frailty, showing promising potential as a prognostic tool.

The CGA applied in oncology practice allows the adoption of tailored treatment according to the frailty degree and patient’s needs and goals [35]. Indeed, geriatric patients with cancer often value the quality of life more than its prolongation [36].

Scores commonly used in oncology for the general population, such as the Karnofsky PS or ECOG PS, are not entirely effective in estimating the frailty of older subjects as they are one-dimensional tools that only examine physical performance [20]. Older adults with cancer, instead, often have multidomain GA impairment that is not easily detectable with standard oncological evaluation [20].

The higher the number of domains impaired, the higher the risk of developing complications due to the treatment, a decline in performance status, a worsening of quality of life, and poorer survival [37].

Our tool composed of the G8 questionnaire, Clinical Frailty Scale, and hand grip test is effective in providing an exhaustive and fast evaluation of all CGA’s domains explored from multiple points of view: self-reported by the patient with the G8 questionnaire, clinical assessment by physicians with the CFS, and the objective measurement of a physical parameter (muscle function) by the HGS.

CGA evaluation is crucial because it improves disease-related outcomes, increases treatment efficacy, minimizes chemotherapy-induced toxicity, and decreases the risk of falls among cancer patients. Furthermore, when the assessment of cancer patients is implemented with CGA, a reduction in morbidity, mortality, and re-hospitalization rate was observed in various reports [18].

In this regard, MOFS demonstrated an excellent ability to comprehensively detect, estimate, and stratify frailty as demonstrated by the strong inverse linear correlation observed with MPI, a well-known benchmark in geriatric multidimensional assessment, even better than G8 screening performed alone.

Despite the proven importance of CGA, only a minority of physicians and health professional operators recognize its utility and the real benefits of frailty screening in guiding treatment decisions and improving the quality of care in older adults [3].

The implementation of CGA in older adults with cancer has been difficult due to the heterogeneity of the available screening tools, poor training in their application, and the lack of a single, efficient, and rapid algorithm for clinical practice [20].

As far as this problem is concerned, the MOFS also proves to be an easy and rapid tool to perform with an execution time lower than the MPI and slightly higher than G8, considering that CFS can be assessed by the physician during the administration of the latter and subsequent measurements of HGS take about two minutes only.

In our opinion, the promising role of MOFS in daily oncology practice lies in the reliability of its three constitutive items and in how they are combined in a comprehensive and complementary manner that fully assesses the various frailty domains.

Thus, as highlighted by van Walree et al., G8 has an optimal diagnostic performance with strong sensitivity and good specificity [20]. In addition, Kanesvaran et al. showed in 249 older cancer patients how hand grip strength was significantly associated with frailty and overall survival [38]; Velghe et al. observed in a cohort of older adults with hematological malignancies that hand grip strength was able to predict an abnormal CGA [39]. Kerschbaumer et al. highlighted the prognostic value of CFS in patients undergoing the resection of brain metastasis [40].

The usefulness of various tools associated has already been suggested in the recent literature.

As mentioned above, the screening tools that build up MOFS have demonstrated in various studies not only diagnostic but also prognostic value, albeit in particular cancer settings [20,38,40].

According to the data from our pilot study, the MOFS shows a strong predictive value in 1-year overall survival in different kinds of settings and for different types of cancer, comparable to MPI.

Our study has some limitations. Firstly, with a small sample size, caution must be applied, as the findings might not be transferable to different cohorts of older patients with cancer. Secondly, the single-center design of the study might have reduced the generalizability of our results; notwithstanding, the monocentric investigation allowed an accurate and standardized data collection.

## 5. Conclusions

Overall, our data demonstrate that MOFS is able to integrate and improve G8 in clinical practice, emerging as a new, accurate, and quick-to-use screening tool for stratifying frailty and the risk of mortality in geriatric cancer patients. However, the future application of MOFS in larger multicenter prospective studies is needed to confirm the usefulness of its preferential routine use for stratifying older cancer patients.

## Figures and Tables

**Table 1:** Characteristics of study population development cohort.

Development Cohort	Whole Cohort *n* = 163	Deceased *n* = 21	Alive *n* = 142	*p*-Value
Age mean years (SD)	80.16 (5.86)	83.3 (4.80)	79.9 (5.88)	0.14
MOFS mean (SD)	8.96 (2.49)	6.38 (2.69)	9.23 (2.33)	<0.001
G8 median (IQR)	13 (2.5)	9 (1.625)	13 (3)	<0.001
CFS median (IQR)	3 (2)	4.5 (2.5)	3 (2)	0.006
BMI mean (SD)	26 (5.5)	25.56 (8.05)	26.03 (5.33)	0.79
BADL median (IQR)	6 (1)	6 (2)	6 (1)	0.15
IADL median (IQR)	7 (3)	6 (4.5)	7 (3)	0.12
Hand grip mean Kg (SD)	17.7 (5.3)	14.06 (4.82)	18.15 (5.31)	0.02
SPSMQ median (IQR)	1 (2)	1.5 (2.5)	1 (2)	0.17
MNA median (IQR)	12 (3)	11 (3.5)	12 (3)	0.004
CIRS median (IQR)	2 (1)	3 (1)	2 (1)	0.61
N° of drugs median (IQR)	5 (4)	6 (3)	5 (4)	0.49
SPPB median (IQR)	7 (6)	3 (6)	8 (7)	0.006
ESS median (IQR)	19 (3)	17.5 (5.5)	19 (2)	0.09
MPI mean (SD)	0.27 (0.14)	0.41 (0.20)	0.26 (0.14)	0.003
GDS median (IQR)	4 (4)	4.5 (4)	4 (4)	0.91
ECOG PS median (IQR)	1 (1)	2 (0.75)	1 (1)	0.02

Continuous variables are expressed as mean SD or median with IQR properly. MOFS indicates Multidimensional Oncological Frailty Scale; CFS, Clinical Frailty Scale; BMI, Body Mass Index; BADL, Basic Activities of Daily Living; IADL, Instrumental Activities of Daily Living; SPSMQ, Short Portable Mental Status Questionnaire; MNA, Mini Nutritional Assessment; CIRS, Cumulative Illness Rating Scale; SPPB, Short Physical Performance Battery; ESS, Exton Smith Scale; MPI, Multidimensional Prognostic Index; GDS, Geriatric Depression Scale; ECOG PS, Eastern Cooperative Oncology Group Performance Status.

**Table 2:** Characteristics of study population validation cohort.

Validation Cohort	Whole Cohort *n* = 70	Deceased *n* = 15	Alive *n* = 55	*p*-Value
Age mean years (SD)	78.6 (6.6)	80.8 (5.6)	78.2 (6.7)	0.26
Sex F (%)	42 (60)	10 (70)	21 (38)	0.03
MOFS mean (SD)	9.23 (3.45)	5.56 (3.53)	9.84 (3.05)	<0.001
G8 median (IQR)	12 (5.375)	7.25 (3.750)	12.75 (4.625)	<0.001
CFS median (IQR)	3 (3)	6.5 (1.750)	3 (2)	<0.001
BADL median (IQR)	6 (1)	5 (4.75)	6 (1)	0.003
IADL median (IQR)	8 (3)	0 (7.25)	8 (2)	<0.001
Hand grip mean Kg (SD)	19.4 (7.07)	14.7 (6.69)	20.24 (6.86)	0.02
BMI mean (SD)	24.9 (4.9)	23.47 (7.8)	25.38 (5.6)	0.30
SPSMQ median (IQR)	1 (2.75)	5 (5)	1 (1)	<0.001
MNA median (IQR)	11 (3)	4 (2.5)	11 (3.5)	<0.001
CIRS median (IQR)	2 (1)	3.5 (1.75)	2 (2)	0.02
N° of drugs median (IQR)	5 (5)	7 (2.75)	5 (5)	0.07
SPPB median (IQR)	6 (5)	2 (5)	7 (6)	0.006
ESS median (IQR)	19 (3)	14.5 (4.5)	19.5 (2)	<0.001
MPI mean (SD)	0.25 (0.30)	0.57 (0.18)	0.25 (0.17)	<0.001
GDS median (IQR)	3 (4)	4 (4)	3 (4)	0.91

Continuous variables are expressed as mean SD or median with IQR properly. MOFS indicates Multidimensional Oncological Frailty Scale; CFS, Clinical Fralty Scale; BADL, Basic Activities of Daily Living; IADL, Instrumental Activities of Daily Living; SPSMQ, Short Portable Mental Status Questionnaire; MNA, Mini Nutritional Assessment; CIRS, Cumulative Illness Rating Scale; SPPB, Short Physical Performance Battery; ESS, Exton Smith Scale; MPI, Multidimensional Prognostic Index; GDS, Geriatric Depression Scale.

**Table 3:** Type of cancer validation cohort.

Type of Cancer	*n* = 70
Colorectal (%)	17 (24.2)
Ovarian (%)	12 (17.1)
Lung (%)	11 (15.7)
Breast (%)	10 (14.2)
Gastric (%)	5 (7.1)
Pancreatic (%)	4 (5.7)
Hematological (%)	3 (4.3)
Genitourinary (%)	3 (4.3)
Esophageal (%)	2 (2.9)
Sarcoma (%)	1 (1.5)
Melanoma (%)	1 (1.5)
GIST (%)	1 (1.5)

## Data Availability

Restrictions apply to the availability of these data. Data were obtained from the Multidisciplinary Breast Center Scientific Committee and are available from the authors with the permission of the Multidisciplinary Breast Center Scientific Committee.
